# Resynchronisation efficacy of pacing strategies depends on the assessment metric: a computational study

**DOI:** 10.3389/fphys.2026.1818421

**Published:** 2026-07-28

**Authors:** Mohammadreza Kariman, Christoph M. Augustin, Gernot Plank

**Affiliations:** 1 Gottfried Schatz Research Center, Medical Physics and Biophysics, Medical University of Graz, Graz, Austria; 2 BioTechMed-Graz, Graz, Austria

**Keywords:** bundle branch block, cardiac resynchronization therapy, computational modelling, conduction system pacing, resynchronization efficacy

## Abstract

**Background:**

Current evaluation of ventricular resynchronisation relies largely on ECGbased metrics, which do not fully capture the complexity of electrical synchrony. Reliance on a single clinical metric provides limited insight into the restoration of the intrinsic physiological activation sequence. To address this, we employed high-fidelity computational simulations using a multiple-metrics framework to mechanistically compare established pacing strategies.

**Methods:**

A high-resolution, anatomically and biophysically detailed four-chamber human model was used to compare the resynchronisation efficacy of conduction system pacing and biventricular pacing under focal bundle branch block. Outcomes were evaluated using: (1) ECG markers (QRS duration, Hisio-ventricular interval, V6-V1 interpeak interval, V6 R-wave peak time); (2) high-density activation maps; and (3) ventricular activation velocity, a novel metric of depolarisation dynamics.

**Results:**

Simulation results showed that pacing efficacy depended on the chosen metric. biventricular pacing improved ECG markers (e.g., QRS 120 to 90 ms) and activation velocity, but activation maps deviated most from physiological patterns. conduction system pacing produced maps resembling normal sinus rhythm, though velocity profiles showed residual dyssynchrony, highlighting that improvement on one metric did not guarantee improvement on another.

**Conclusion:**

These findings show that pacing efficacy depends on the evaluation metric and that single-metric assessment cannot capture the complex dynamics of ventricular resynchronisation. Patient-specific computational modelling offers a promising approach for non-invasive, multi-modal in silico evaluation.

## 1 Introduction

Heart failure remains a major and growing global health burden, and its progressive nature continues to drive significant clinical and economic challenges worldwide (Roger, 2013; Darvish et al., 2025). Over recent decades, multiple ventricular pacing strategies have been developed to mitigate the electromechanical disturbances characteristic of advanced heart failure. Among these, cardiac resynchronization therapy (CRT) delivered through biventricular pacing (BiVP) has consistently demonstrated improvements in electrical coordination, cardiac performance and clinical outcomes, including reductions in both mortality and hospitalisation (Cleland et al., 2005; Tang et al., 2010; Zareba et al., 2011; Glikson et al., 2021).

Yet despite these established benefits, a considerable proportion of patients fail to exhibit meaningful clinical improvement (Ruschitzka et al., 2013; Vernooy et al., 2014), underscoring the need for alternative approaches such as conduction system pacing (CSP), which seeks to restore a more physiological pattern of ventricular activation and overcome limitations associated with BiVP (Vijayaraman et al., 2021; Vijayaraman and Batul, 2022; Kaddour and Burri, 2023; Glikson et al., 2025). Central to understanding the benefit of any pacing strategy is the effectiveness of electrical resynchronisation, which encompasses not only acceleration of ventricular activation but also restoration of a physiological activation sequence; however, current clinical metrics predominantly reflect global timing and incompletely capture the latter. While QRS duration (QRSd) shortening remains the most widely used surrogate for electrical resynchronisation (Jastrzębski et al., 2019; Gerra et al., 2025), accumulating evidence indicates that it does not fully capture the complexity of ventricular activation. Recent comparative work using ECG, vectorcardiography, and ultrahigh-frequency ECG has further demonstrated that the assessment of dyssynchrony is highly dependent on the method and metric used (Rijks et al., 2024), raising questions regarding the adequacy of any single measure as a standalone indicator.

Computational cardiac modelling offers a rigorous mechanistic framework for examining dyssynchrony at a level of control not achievable clinically. By providing anatomically detailed, electromechanically coupled simulations that extend beyond what can be resolved clinically (Lee et al., 2018; Strocchi et al., 2022; Meiburg et al., 2023), these models enable controlled comparison of pacing strategies, clarify modality-specific strengths and limitations, and support systematic evaluation of emerging physiological pacing approaches (Strocchi et al., 2025). Critically, computational frameworks can identify mechanistic insights translatable to therapeutic protocols and medical device programming strategies (Bhagirath et al., 2024; Strocchi et al., 2024; Trayanova et al., 2024).

In this study, we employed an anatomically and biophysically detailed computational model of the human four-chamber heart, incorporating realistic representation of the cardiac conduction system, which allowed for systematic and controlled evaluation of both conventional pacing strategies and CSP, including left bundle branch pacing (LBBP) and right bundle branch pacing (RBBP), under bundle branch block conditions. Beyond conventional ECG-derived markers, we complemented our analysis with a mechanistic evaluation of ventricular activation patterns and introduced a novel metric, the *activation velocity*, to provide a more nuanced assessment of electrical resynchronisation.

Our findings demonstrate that the apparent efficacy of a given pacing modality is highly dependent on the metric used for evaluation; reliance on a single-domain measure such as QRSd alone may fail to capture the full extent of ventricular resynchronisation, highlighting the benefits of multimodal assessment in silico. By adopting a translational research perspective, this work identifies the limitations of single-metric approaches and demonstrates how computational modelling may serve as a tool to support integrative assessment of pacing strategies in silico.

## 2 Methods

### 2.1 Patient-specific anatomical model

A patient-specific anatomical model of heart and torso of a 45-year-old male subject was derived from magnetic resonance imaging (MRI) (Ethics approval: Medical University of Graz, EKNr: 24–126 ex 11/12). The image-based heart and torso geometry was generated using a previously described anatomical twinning workflow, in which clinically acquired MRI data were segmented to derive the subject-specific computational anatomy (Gillette et al., 2021).

**Figure 1:**
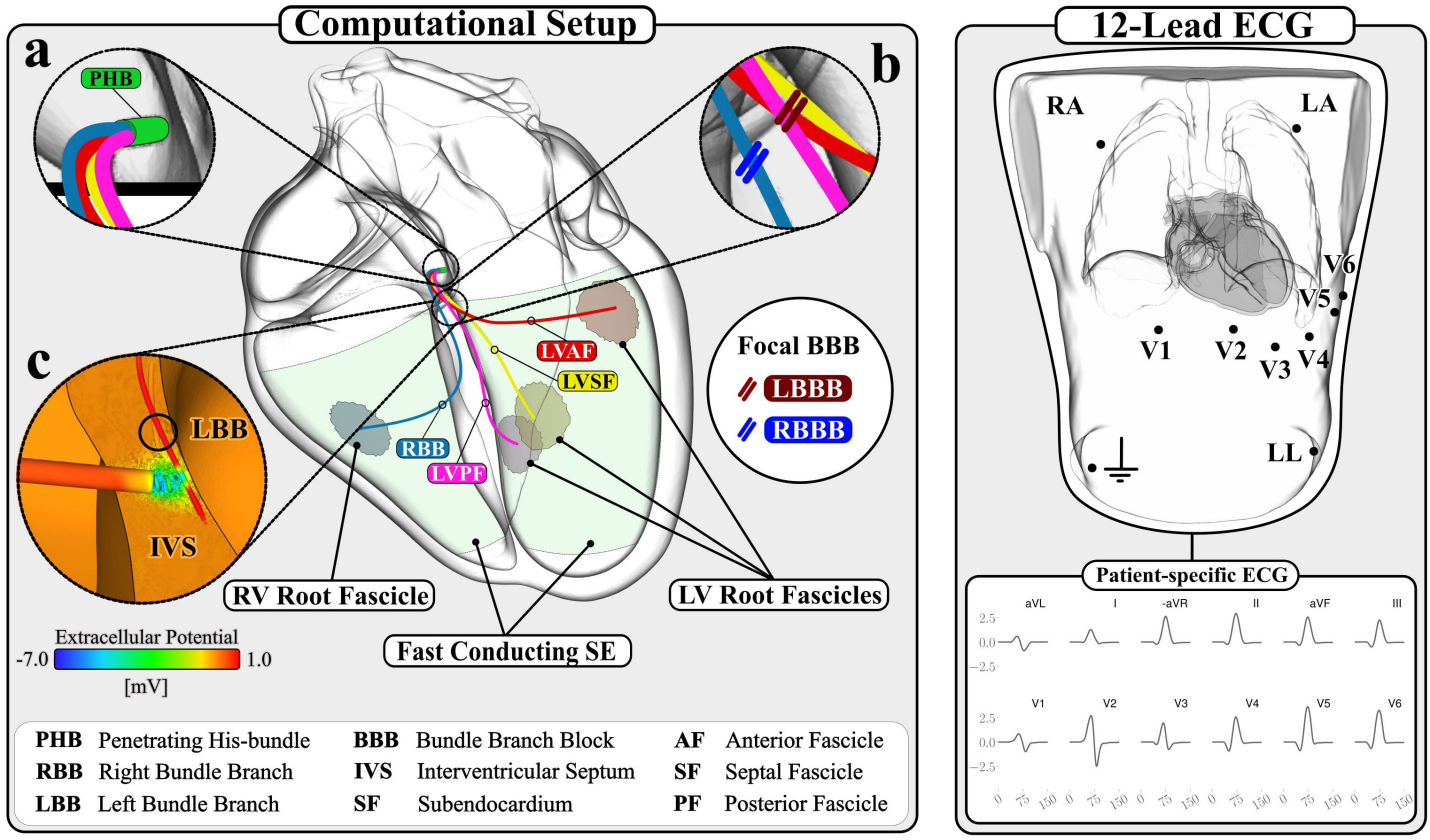
Left: Anatomical model of the four-chamber human heart with a detailed ventricular conduction system. The LBB comprises three fascicles – anterior (red), septal (yellow), and posterior (pink) – and the RBB (blue), all originating from the penetrating His-bundle **(a)**. Bundle branch block conditions are shown in panel **(b)** The electric field generated by the pacing lead from the extracellular potential **(c)**. Right: The corresponding 12-lead ECG derived from the model under normal sinus rhythm (NSR).

Ventricular fibre orientation was assigned using a rule-based approach (Bayer et al., 2012) assuming fibers rotating linearly from 60° at the endocardium to −60° at the epicardium, and sheet angles from −65° to 25°, respectively. A high-fidelity three-dimensional anatomical representation of the ventricular conduction system was constructed in accordance with published anatomical descriptions (Stephenson et al., 2017; Padala et al., 2021; Chen et al., 2025), comprising penetrating His-bundle giving rise to cable-like right and left bundle branches (**Figure 1**).

The anterior and posterior left bundle branch (LBB) fascicles were positioned to terminate near the corresponding papillary muscle regions, whereas the septal fascicle spanned between these two distributions (Elizari, 2017). The right bundle branch (RBB) was modelled to course through the septum toward the moderator band and to terminate near the anterior papillary muscle region (Shenoy et al., 2016; Lee and Hur, 2019), together producing four major early endocardial activation regions. Focal conduction blocks were imposed at proximal segments of the respective bundle branches to represent left bundle branch block (LBBB) or right bundle branch block (RBBB) conditions.

### 2.2 Electrophysiological simulations

A hybrid approach was employed to simulate ventricular electrophysiology. Initiation of propagation by pacing was simulated using a bidomain model (Vigmond et al., 2008). For this sake, a geometrically detailed model of a pacing lead (Medtronic, 2023) widely used for CSP was incorporated using *NumeriCor Studio* (NumeriCor GmbH, Graz, Austria) to predict the volume of myocardial and fascicular tissue that is directly captured by the electric field generated by the implanted lead, using the same lead model for all pacing strategies. After stimulation, action potential propagation was simulated using a computationally efficient reaction-eikonal model (Neic et al., 2017).

The ventricular myocardium was modelled as an orthotropic medium with conduction velocities of 0.6m*/*s, 0.4m*/*s, and 0.2m*/*s along the fibre, sheet, and sheet-normal directions, respectively. The distal fast-conducting subendocardial layer was assumed isotropic and was introduced as a functional surrogate of rapid Purkinje-mediated endocardial activation rather than as an explicit anatomical reconstruction of the terminal Purkinje network. Consistent with this simplification, all regions representing specialised ventricular conduction tissue, including the fascicles and the fast-conducting subendocardial layer, were assigned the same conduction velocity of 2.0m*/*s (Gillette et al., 2021). Conductivities were calibrated for the given mesh resolution to reproduce the prescribed conduction velocities. In the ventricular myocardium, the resulting intracellular and extracellular conductivities were *σ*_i_,_f_ = 0.58 S*/*m and *σ*_e_,_f_ = 2.11 S*/*m along the fibre direction, *σ*_i_,_s_ = 0.21 S*/*m and *σ*_e_,_s_ = 2.67 S*/*m along the sheet direction, and *σ*_i_,_n_ = 0.048 S*/*m and *σ*_e_,_n_ = 0.61 S*/*m in the sheet-normal direction. In the fast-conducting subendocardial layer, isotropic conductivities of *σ*_i_ = 1.07 S*/*m and *σ*_e_ = 3.91 S*/*m were used. Cellular dynamics in ventricular myocardium and conduction system were modelled using the ten Tusscher (Ten Tusscher and Panfilov, 2006) and Stewart model (Stewart et al., 2009), respectively. Simulations were executed using CARPentry software (Vigmond et al., 2008; Plank et al., 2021).

The torso model incorporated exact image-based ECG electrode locations to compute a standard 12-lead electrocardiogram (ECG) from the ventricular electrical sources using a lead field formulation (Gillette et al., 2021; Pezzuto et al., 2021). The conduction system was calibrated to closely match the ECG recorded for this subject **Figure 1** by adjusting the location of the fascicular terminals within the ventricular subendocardium. Recorded and simulated ECGs were processed with a 150Hz low-pass filter, a 50Hz notch filter, and a 0.05Hz high-pass filter.

### 2.3 Metrics for activation and synchronicity

Ventricular activation of the left and right ventricle is a spatiotemporally distributed process for which no single, universally applicable measure of synchrony exists. Thus, the assessment of a pacing strategy for improving efficacy of chamber activation and synchronicity under conditions such as LBBB or RBBB critically depends on the metrics used. To characterize optimality of a pacing strategy we use distinct metrics comprising both clinically observable ECG-based markers as well as purely computational markers that best reflect intrinsic chamber behaviors, to measure the efficacy of pacing in improving activation sequence and inter-ventricular synchronization.

#### 2.3.1 Clinical ECG markers

**Figure 2:**
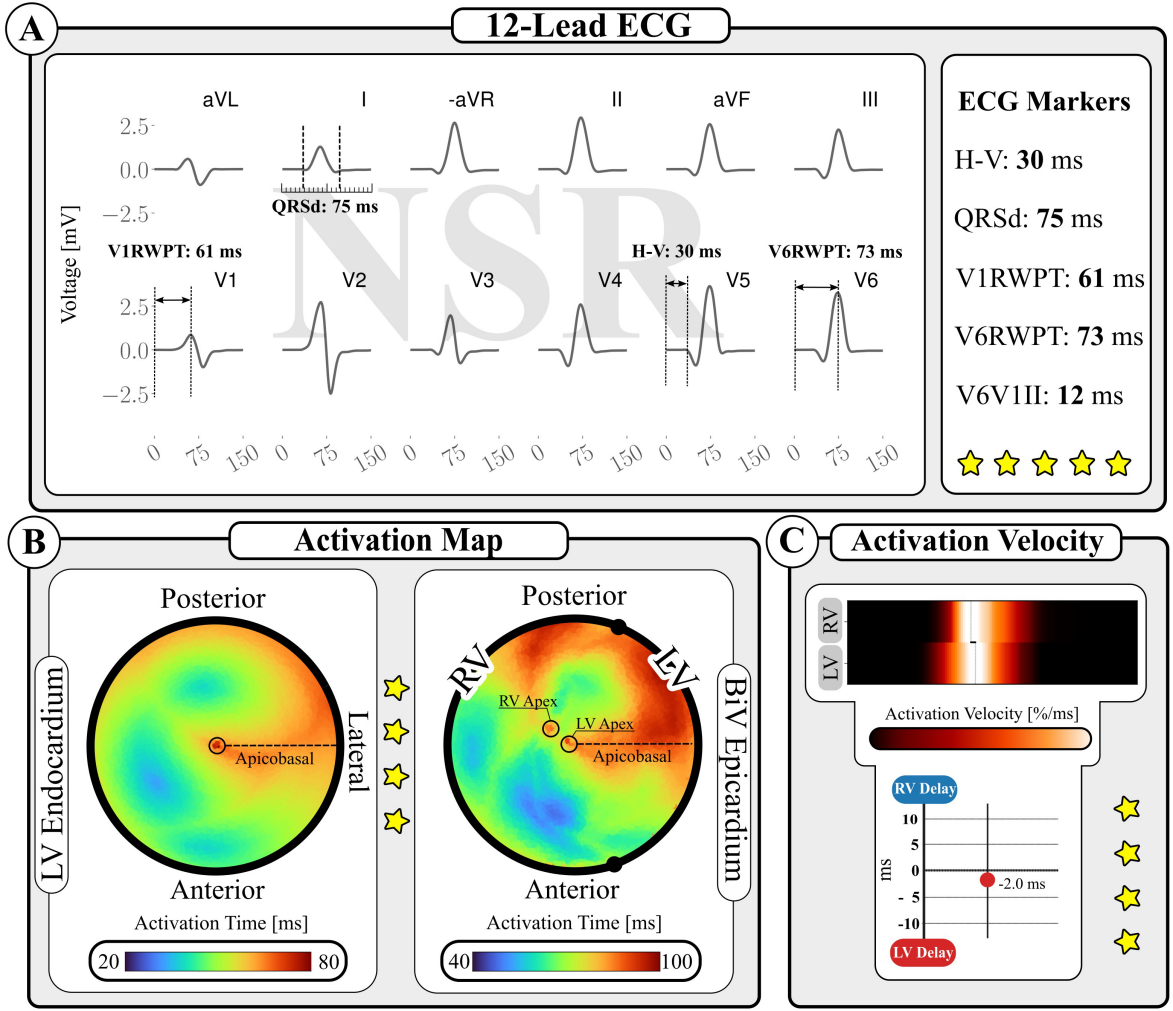
Metrics used to assess ventricular synchrony: **(A)** ECG-derived markers from the 12-lead ECG, **(B)** left ventricular endocardial and biventricular epicardial activation maps, and **(C)** activation velocity profiles of the left and right ventricles.

Common ECG-based clinical indices providing a global view on activation and synchronicity were used. These included QRSd, His potential to onset of QRS represented by the Hisio-ventricular (H-V) interval, interventricular delay (IVD) reflected by V6-V1 interpeak interval (V6V1II), and right ventricle (RV) and left ventricle (LV) activation time as measured by V1 R-wave peak-time (V1RWPT) and V6 R-wave peak-time (V6RWPT) respectively (**Figure 2A**). All ECG markers were determined computationally from the simulated 12-lead ECG, and reported values were rounded for clarity of presentation.

#### 2.3.2 Activation maps

Three-dimensional ventricular activation maps were converted to two-dimensional high-density bull’s-eye plots leveraging universal ventricular coordinates (Bayer et al., 2018), to represent activation of the LV endocardium and the biventricular epicardium (**Figure 2B**). The synchronicity of paced activation sequences, LAT_p_, is measured then by the similarity to the NSR activation map observed under healthy conditions (**Figure 5a** and **A**), LAT_nsr_, as a difference map, ΔLAT = LAT_p_ − LAT_nsr_, assuming that the NSR activation produces optimal hemodynamic performance.

#### 2.3.3 Activation velocity

IVD is traditionally assessed based on echo-derived mechanical indices (Bax et al., 2004; Van Bommel et al., 2010) or electrophysiological surrogates such as QRSd, which, despite its widespread use for estimating IVD and selecting CRT candidates, remains debated (Mollema et al., 2007; Delgado and Bax, 2011).

Synchronous ventricular contraction requires a matching rate in myocardial recruitment between the ventricles by depolarization wave fronts over the duration of ventricular activation. Mechanistically, recruitment can be quantified by activation velocity defined as the relative myocardial volume depolarizing at each instant in time (**Figure 2C**). The difference in temporal activation velocity profiles between RV and LV termed *activation velocity mismatch (AVM)* provides a dynamic timedependent measure of dyssynchrony beyond static scalar surrogate markers. To assess dyssynchrony over the entire ventricular activation the root mean square error (RMSE) of the AVM profile was calculated, providing a single index of IVD.

### 2.4 Evaluation of pacing strategies

**Figure 3:**
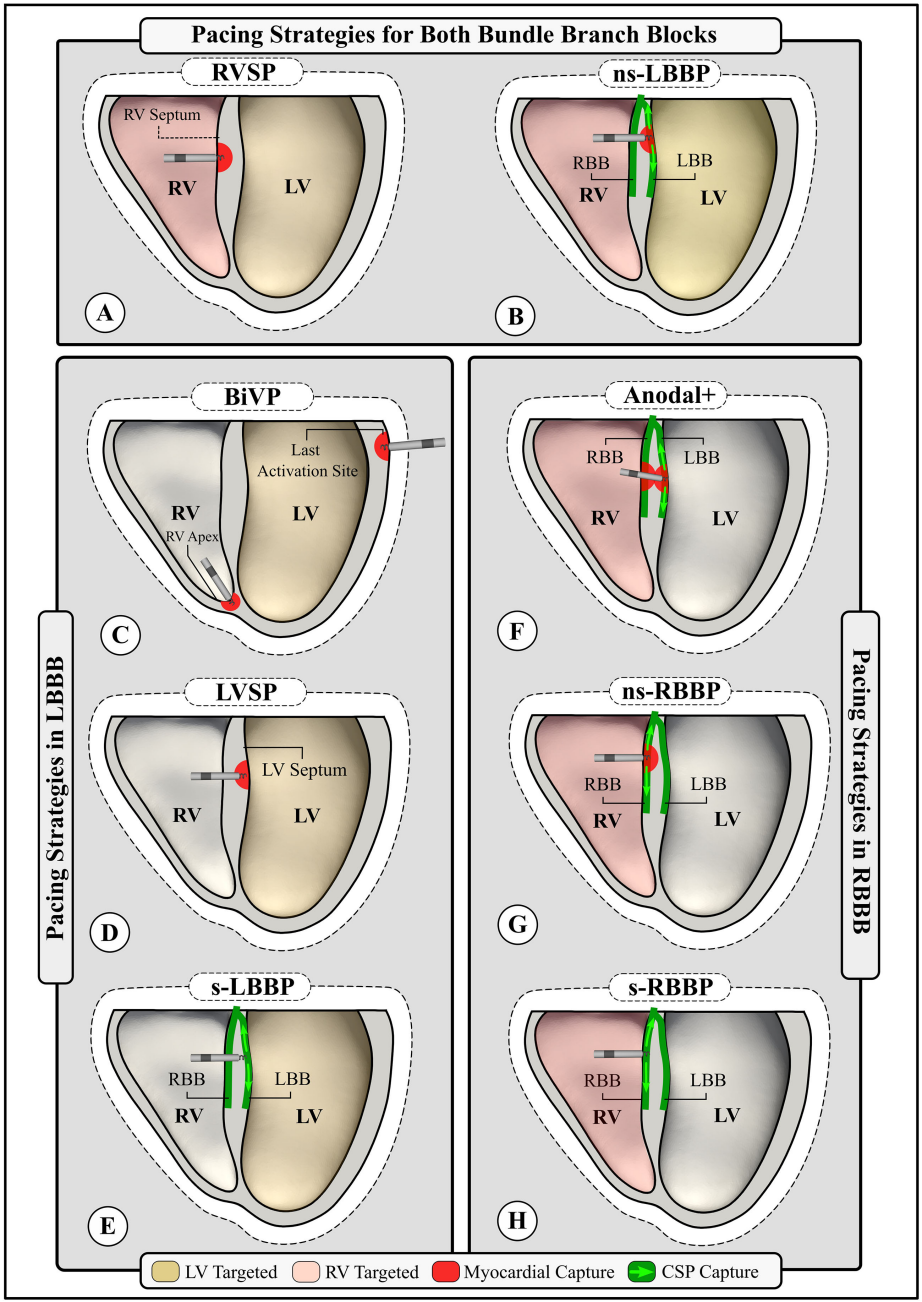
Pacing modalities investigated under LBBB (left: **(C–E)**) and RBBB (right: **(F–H)**). Strategies applicable to both bundle branch block conditions are shown in the top row **(A, B)**, with LBBB-specific and RBBB-specific pacing configurations presented in the corresponding left and right panels.

The efficiency of conventional and CSP pacing strategies under LBBB and RBBB conditions for enhancing activation and synchronicity was assessed using ECG markers, endocardial and epicardial activation maps as well as activation velocity as metrics. Leads were positioned at anatomically realistic implant sites (**Figure 3**; **Table 1**), where timing of pacing pulse delivery were kept constant. The following pacing strategies were considered:

**Table 1:** Overview of the pacing strategies evaluated in this study, including stimulation settings, pacing tip location, and the corresponding capture mode.

Strategy	Threshold	Pacing tip location	Description
RVSP	1.0V@ 0.5ms	Mid-apico-basal RV septum	Myocardial capture
BiVP	1.0V@ 0.5ms	LV lateral epicardial wall; RV apex	Dual-site myocardial capture
LVSP	1.5V@ 1.0ms	LV subendocardium	Myocardial capture
s-LBBP	1.0V@ 0.5ms	Proximal LBB region	Selective LBB capture
ns-LBBP	1.5V@ 1.0ms	Proximal LBB region	LBB plus myocardial capture
Anodal+	5.0V@ 1.0ms	Proximal LBB region	ns-LBBP settings plus RV septal capture
s-RBBP	1.0V@ 0.5ms	Proximal RBB region	Selective RBB capture
ns-RBBP	1.5V@ 1.0ms	Proximal RBB region	RBB plus RV septal myocardial capture

Thresholds denote the pacing output and pulse width used in the simulations for each lead configuration.

- RVSP: Lead positioned at the mid-apico-basal RV septum near the outflow tract, capturing septal myocardium (**Figure 3A**).
- BiVP: Dual-site pacing with the LV lead placed on the lateral epicardial wall within the last activation site (LAS) and the RV lead at the apical septum (**Figure 3C**).
- LVSP: Lead placed deep into the interventricular septum (IVS) to stimulate the LV subendocardium. (**Figure 3D**).
- s-LBBP: Lead advanced deep into the IVS to achieve selective capture of the proximal LBB without IVS engagement (**Figure 3E**).
- ns-LBBP: Similar to selective left bundle branch pacing (s-LBBP), with additional recruitment of adjacent LV sub-endocardial IVS myocardium (**Figure 3B**).
- Anodal+: Variant of ns-LBBP with supplementary capture of RV septal myocardium near the anodal ring electrode (**Figure 3F**).
- s-RBBP: Lead positioned superficially within the RV IVS to selectively recruit the RBB without myocardial capture (**Figure 3H**).
- ns-RBBP: Analogous to s-RBBP, but with additional engagement of adjacent RV septal myocardium near the pacing tip (**Figure 3G**).

## 3 Results

### 3.1 ECG assessment of pacing strategies in LBBB

**Figure 4:**
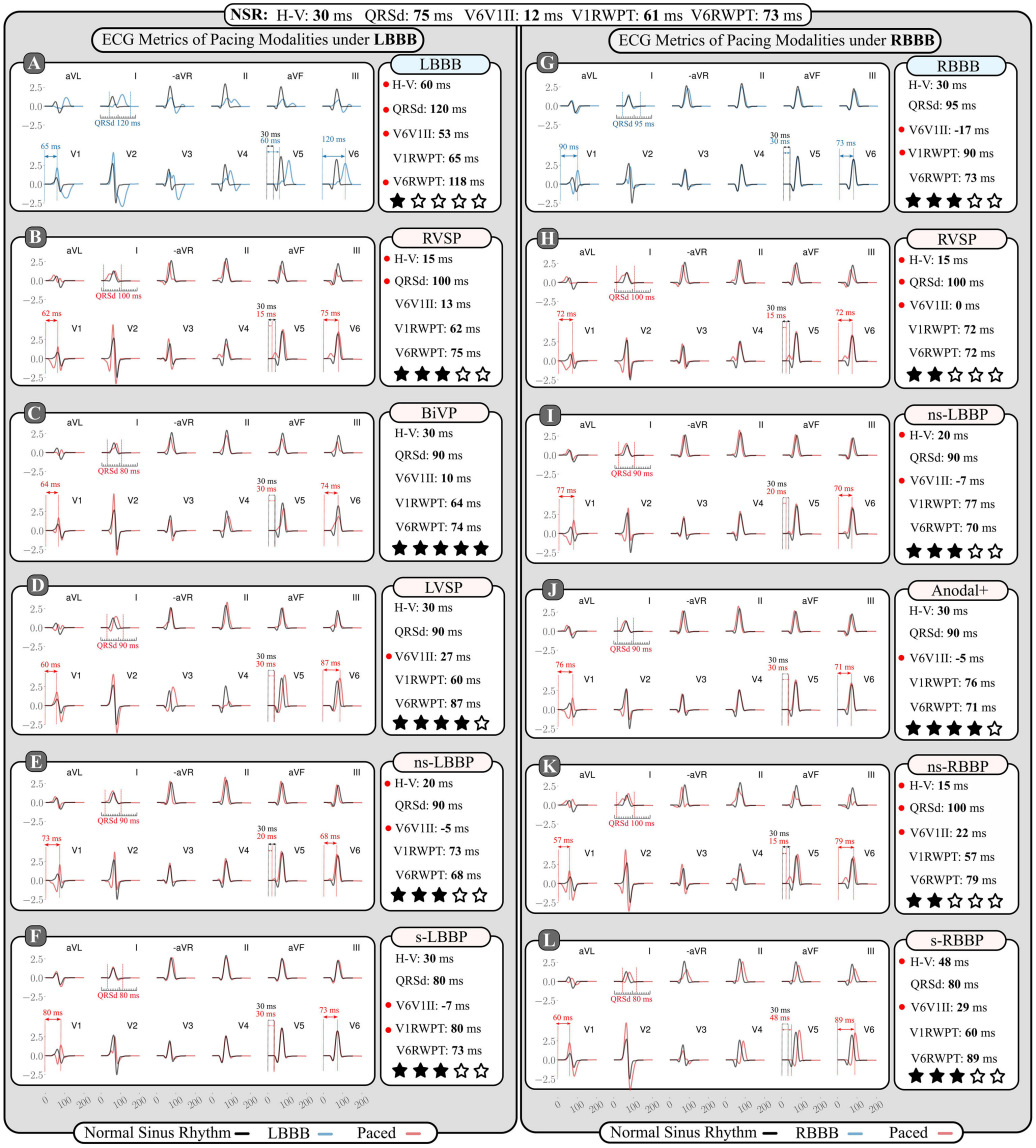
Simulated 12-lead ECGs for selected pacing modalities under LBBB (left panel: **(A–F)**) and RBBB (right panel: **(G–L)**) conditions, with key ECG markers highlighted for each configuration. Red dots next to the ECG markers indicate values exceeding the scoring scheme thresholds. The corresponding star-based rating scheme is detailed in Appendix Table A.1.

Under LBBB (**Figure 4A**), all pacing strategies improved electrical synchrony relative to baseline, albeit with distinct differences in their corrective capacity. The most pronounced QRS narrowing was achieved with s-LBBP, reducing QRSd from 120 ms to 80 ms (**Figure 4F**). non-selective left bundle branch pacing (ns-LBBP), BiVP, and left ventricular septal pacing (LVSP) also resulted in QRS shortening to 90 ms (**Figures 4C–E**), whereas right ventricular septal pacing (RVSP) produced the longest paced QRSd, remaining close to 100 ms (**Figure 4B**).

Near-physiological H-V intervals were achieved under s-LBBP, BiVP, and LVSP. In contrast, the H-V interval was shortened under ns-LBBP and RVSP by approximately 10 ms and 15 ms, respectively, relative to NSR, consistent with a degree of myocardial capture.

RV activation timing, assessed by V1RWPT, was preserved close to baseline under RVSP, BiVP, and LVSP. By contrast, V1RWPT was prolonged under s-LBBP and ns-LBBP, reaching 80 ms and 73 ms, respectively, reflecting retrograde activation of the RBB. All pacing modalities substantially accelerated left ventricular activation, with V6RWPT decreasing from 120 ms under baseline LBBB to values below 90 ms. This effect was most pronounced with LBBP, achieving V6RWPT of 73 ms under s-LBBP and 68 ms under ns-LBBP, whereas LVSP exhibited the longest V6RWPT at 87 ms, indicating incomplete recruitment of LBB fibres and residual IVD attributable to myocardial capture (**Figure 4D**).

Interventricular delay, quantified by V6V1II, improved substantially under RVSP and BiVP, decreasing from 55 ms in baseline LBBB to 13 ms and 10 ms, respectively, values close to NSR (**Figures 4B, C**). In contrast, V6V1II was reversed under LBBP, reaching -7 ms with s-LBBP and -5 ms with ns-LBBP, representing a larger deviation from the NSR value of 12 ms and indicating RV-late activation. Both LBBP modes produced a characteristic RBBB-like pattern in lead V1, with *r*^′^*/R*^′^ deflections consistent with retrograde conduction through the RBB (**Figures 4E, F**).

### 3.2 ECG assessment of pacing strategies in RBBB

Under RBBB, all evaluated pacing strategies improved electrical synchrony to varying degrees, as reflected by modification of the terminal R-wave morphology in lead V1, although the magnitude and pattern of improvement differed between modalities. The most pronounced QRS narrowing was observed under selective right bundle branch pacing (s-RBBP), with QRSd reduced to 80 ms (**Figure 4L**), compared with 100 ms under non-selective right bundle branch pacing (ns-RBBP); however, neither strategy restored QRS duration to NSR values.

Preservation of the apparent H-V interval was most evident under ns-LBBP plus anodal capture (Anodal+), which maintained values comparable to NSR (**Figure 4J**). In contrast, s-RBBP prolonged the H-V interval to 48 ms, whereas ns-RBBP markedly shortened it to 15 ms (**Figures 4K, L**).

Correction of RV activation timing, assessed by V1RWPT, was most effective under RBBP, with values of 57 ms and 60 ms for ns-RBBP and s-RBBP, respectively (**Figures 4K, L**). RVSP, ns-LBBP, and Anodal+ also shortened V1RWPT from 90 ms under baseline RBBB to 72 ms, 77 ms, and 75 ms, respectively (**Figures 4H–J**), although residual delay persisted.

Left ventricular activation timing, reflected by V6RWPT, was prolonged under RBBP, consistent with retrograde recruitment of the LBB, whereas under the remaining pacing modalities it was preserved close to NSR. Interventricular delay, quantified by V6V1II, improved most under RVSP and ns-RBBP, shifting from -17 ms under baseline RBBB to 0 ms and 22 ms, respectively, values closest to NSR. The remaining strategies – ns-LBBP, Anodal+, and s-RBBP – were associated with larger residual V6V1II of -7 ms, -3 ms, and 29 ms, respectively.

### 3.3 Activation patterns of pacing strategies in LBBB

**Figure 5:**
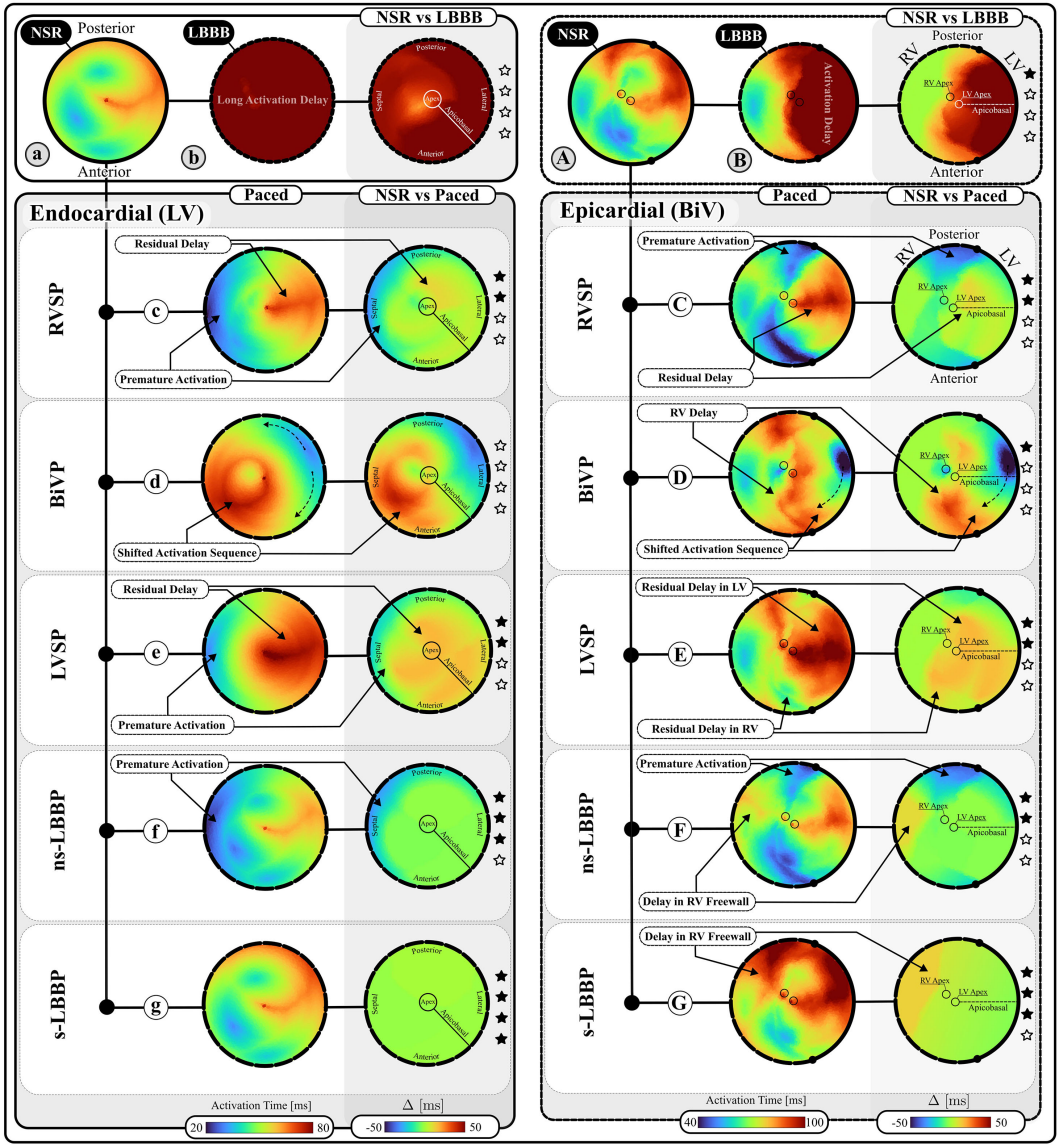
Activation sequence maps of the LV endocardium **(a–g)** and biventricular epicardium **(A–G)** during NSR, LBBB, and selected pacing modalities applied under LBBB conditions. The corresponding star-based rating scheme is detailed in Appendix Table A.2.

In LBBB (**Figure 5b** and **B**), the efficacy of pacing strategies in restoring physiological ventricular activation varied. s-LBBP most effectively corrected the LV activation delay, producing a propagation pattern closely resembling NSR (**Figure 5g** and **G**). ns-LBBP similarly restored LV activation but induced premature basal septal activation (**Figure 5f** and **F**) and advanced posterior RV activation due to local myocardial capture, while both forms exhibited delayed lateral RV wall activation through retrograde RBB conduction (**Figure 5F, G**). RVSP improved LV activation by engaging local septal myocardium, advancing both anterior and posterior RV walls, though residual LV delay remained (**Figure 5C**). LVSP produced only minor premature basal septal activation compared to RVSP and caused greater delay in the LV lateral wall (**Figure 5e** and **E**) due to incomplete engagement of LBBB fascicles and more proximal and posterior lead placement; RV activation was modestly affected, with posterior wall advancement and anterior wall delay (**Figure 5E**). BiVP markedly deviated from physiological activation: the LAS shifted to the anteroseptal region (**Figure 5d** and **D**), and apical lead placement inverted the RV apex to become the new earliest activation site (**Figure 5D**). Overall, LV activation was best restored with selective LBBP, followed by nonselective LBBP, LVSP, and RVSP, with BiVP showing the least physiological LV pattern. RV activation remained closest to normal under BiVP and LVSP, whereas RVSP and nonselective LBBP caused premature posterior RV activation, with both forms of LBBP also delaying the lateral RV wall via retrograde RBB conduction.

### 3.4 Activation patterns of pacing strategies in RBBB

**Figure 6:**
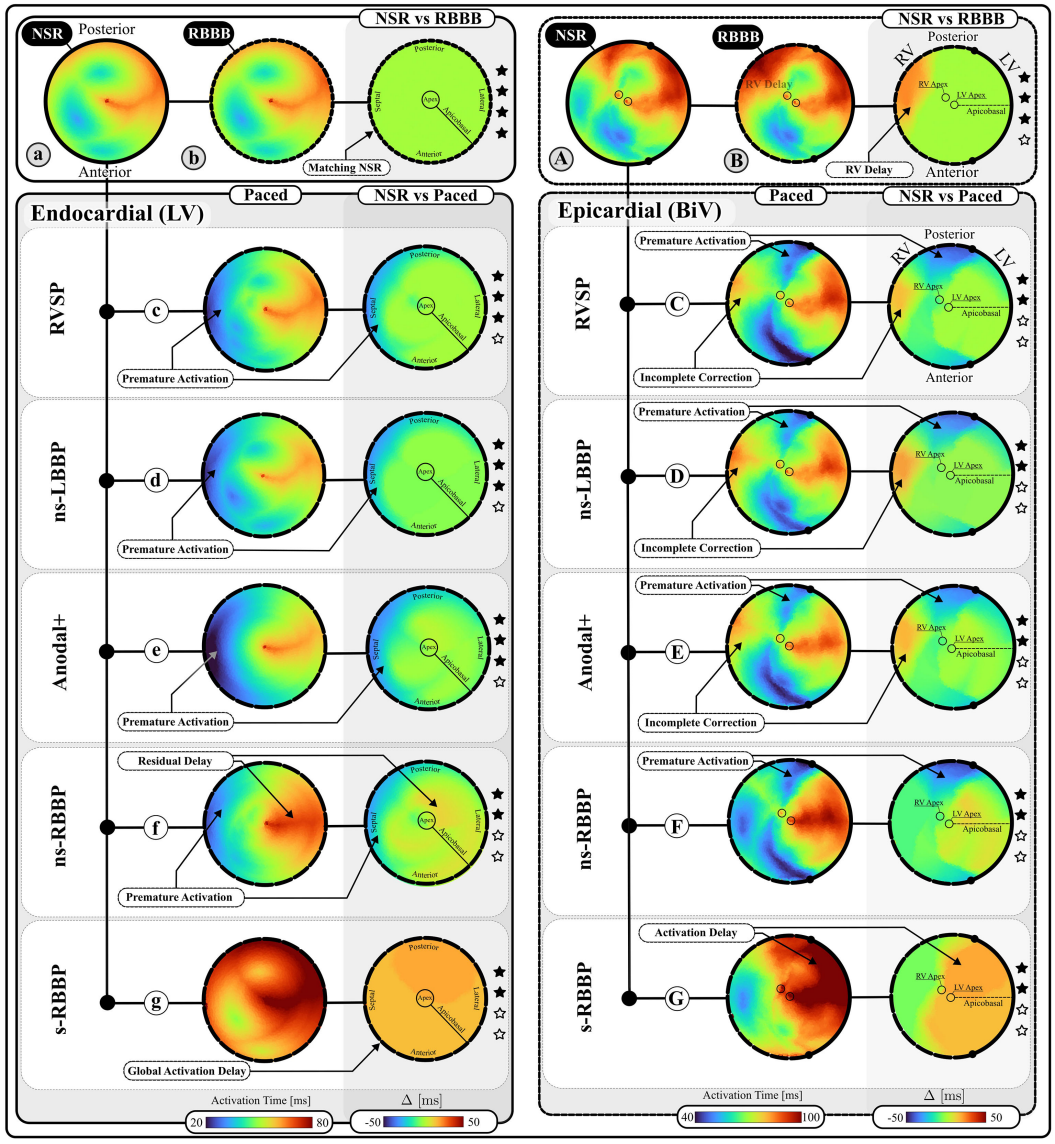
Activation sequence maps of the LV endocardium **(a–g)** and biventricular epicardium **(A–G)** during NSR, RBBB, and selected pacing modalities applied under RBBB conditions. The corresponding star-based rating scheme is detailed in Appendix Table A.2.

In RBBB, pacing strategies demonstrated variable efficacy in correcting RV activation. s-RBBP was the most effective, restoring RV activation to near-physiological patterns, albeit with pronounced global LV conduction delay (**Figure 6g** and **G**). ns-RBBP also improved RV activation, producing partial LV delay, but induced premature excitation of the RV posterior wall (**Figure 6f** and **F**). LBBP, including both Anodal+ and ns-LBBP, was less effective in correcting RV delay, particularly in the free wall, while similarly causing premature posterior RV activation and minimally affecting LV activation; Anodal+ was slightly more effective than ns-LBBP in partially correcting RV lateral wall delay (**Figure 6D, E**). RVSP produced comparable effects to LBBP, partially correcting RV delay with minimal influence on LV activation (**Figure 6c** and **C**).

**Figure 7:**
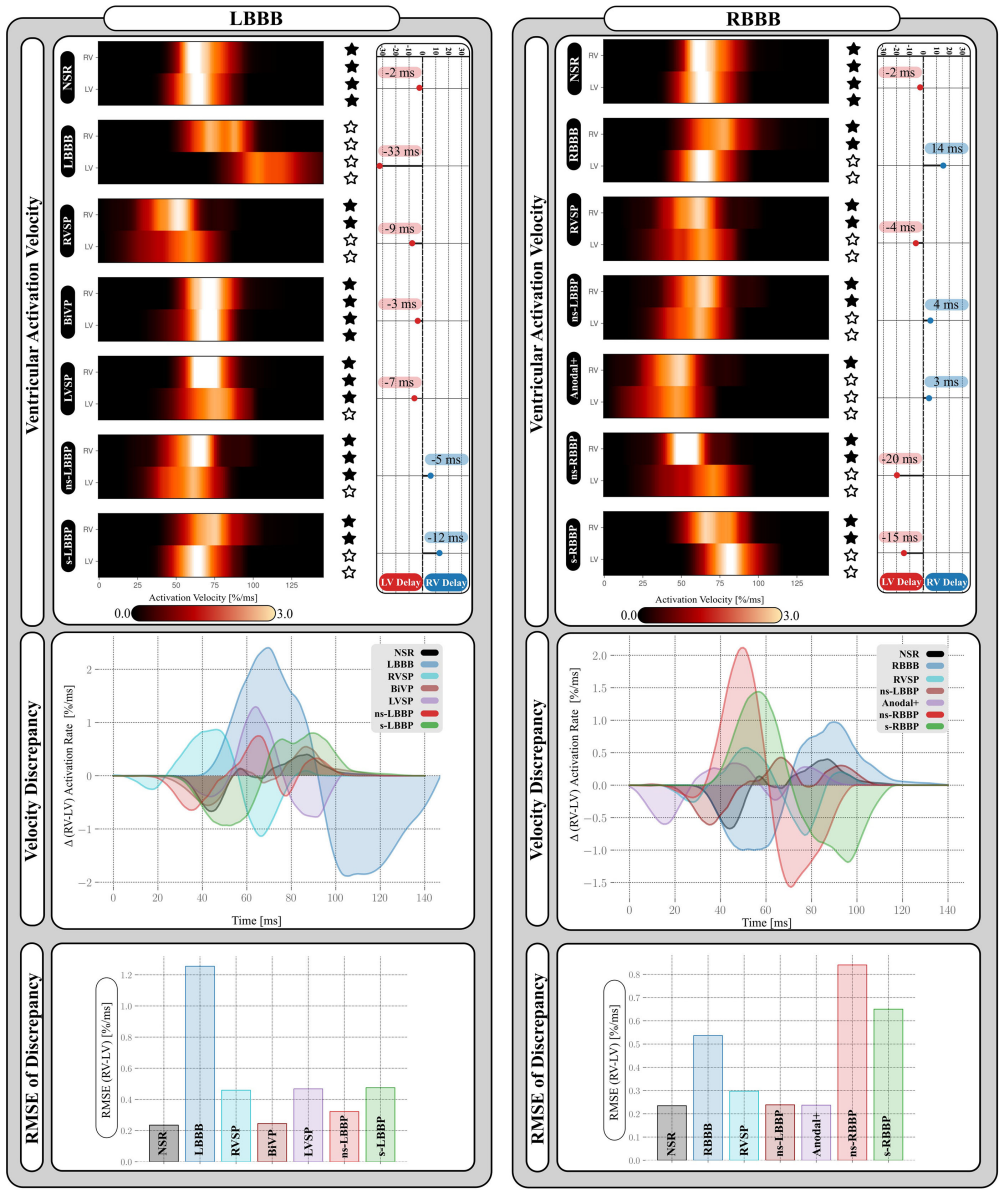
Analysis of activation velocity in the RV and LV during NSR, bundle branch block, and the corresponding pacing modalities (top row). The deviation between RV and LV activation velocities, referred to as discrepancies, is shown in the middle row. The RMSE of these discrepancies is presented in the bottom row. The left panel corresponds to the LBBB condition, and the right panel to the RBBB condition. Temporal dyssynchrony, illustrated in the top panel, was quantified by calculating the interval difference between the peak velocities. The corresponding star-based rating scheme is detailed in Appendix Table A.3.

### 3.5 Activation velocities of pacing strategies in LBBB

**Figure 8:**
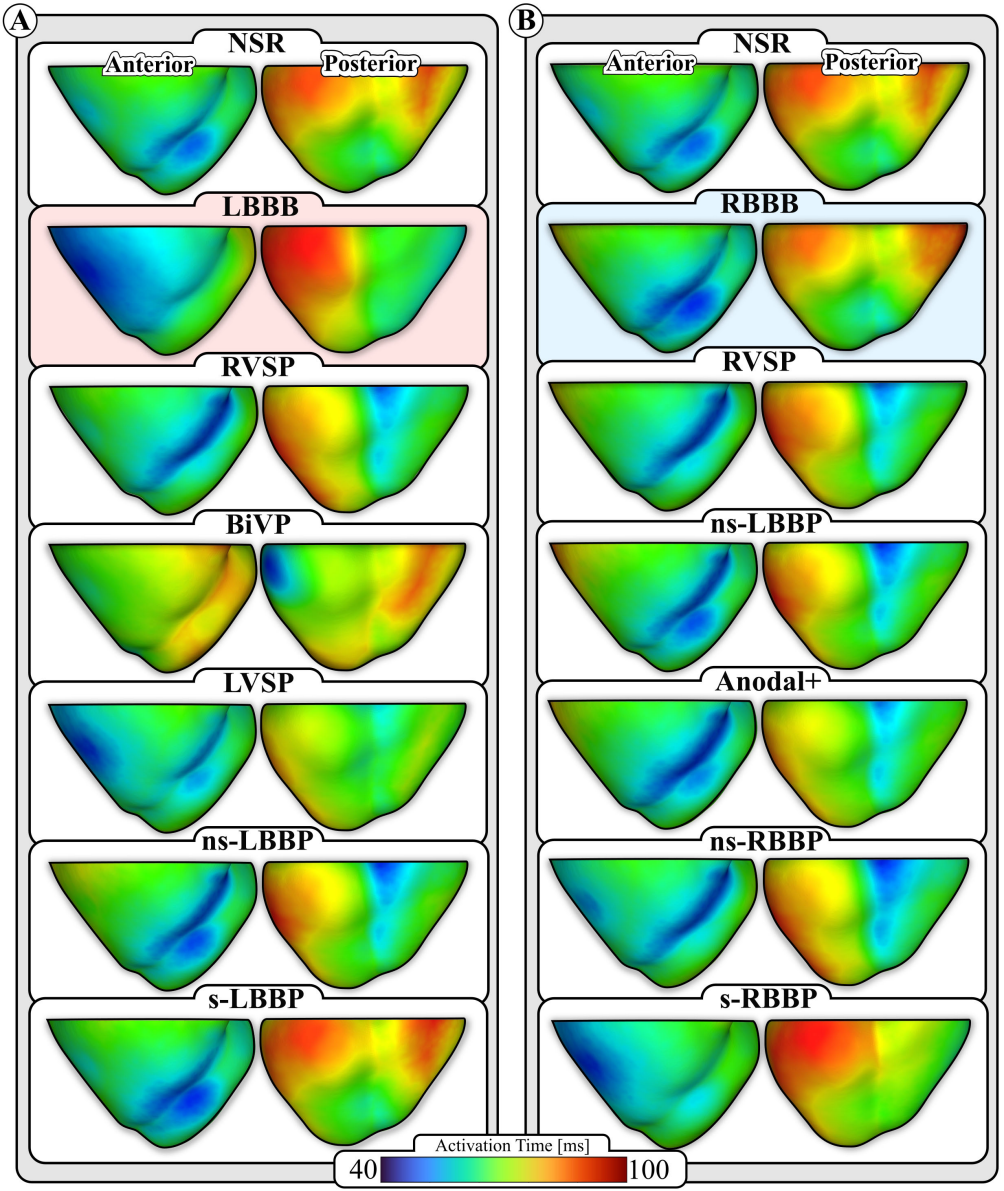
Full 3D ventricular activation patterns for all simulated conditions. Panel **(A)** shows NSR, LBBB, and the pacing strategies applied under LBBB; Panel **(B)** shows NSR, RBBB, and the pacing strategies applied under RBBB. For each condition, activation is shown on the anterior and posterior ventricular surfaces.

Under LBBB, pacing strategies produced distinct effects on ventricular activation velocity profiles (**Figure 8** left panel). RVSP reduced the temporal offset between left and right ventricular fast activation phase (FAP) from –33 to –9 ms, although the RV FAP became prolonged and slower compared with NSR (**Table 2**); nevertheless, the AVM, measured by RMSE, decreased relative to the unpaced LBBB state (**Figure 8** left bottom panel). BiVP most closely reproduced physiological activation velocity, restoring the duration, magnitude, and temporal alignment of the ventricular FAP to values near NSR (**Table 2**) and yielding an AVM very close to baseline. LVSP also improved interventricular synchrony by aligning the FAP, but the LV FAP remained prolonged with decreased magnitude, resulting in persistently elevated AVM compared with normal conduction. Selective and non-selective LBBP generated distinct responses: both reduced the temporal mismatch of the FAP, with greater efficiency under ns-LBBP; in ns-LBBP, the LV FAP magnitude was reduced relative to NSR while the RV profile was nearly preserved, whereas the opposite pattern occurred under selective LBBP (**Figure 8** left panel). Overall, activation velocity resynchronisation was best restored under BiVP, with LVSP and ns-LBBP demonstrating comparable improvements.

**Table 2:** Maximum and mean ventricular activation velocities (%/ms) in LV and RV measured under NSR, LBBB, and corresponding pacing modalities, corresponding to **Figure 7**.

	LV		RV	
Condition	Mean	Max	Mean	Max
NSR	1.60	3.20	1.50	3.30
LBBB	1.25	2.30	1.25	2.70
RVSP	1.20	2.40	1.10	2.90
BiVP	1.25	3.50	1.25	3.30
LVSP	1.30	2.60	1.40	3.60
ns-LBBP	1.25	2.70	1.10	3.25
s-LBBP	1.50	3.10	1.10	2.70

Maximum velocity denotes the peak activation rate reached during depolarisation, whereas mean velocity represents the average activation rate over the entire depolarisation period.

### 3.6 Activation velocities of pacing strategies in RBBB

Under RBBB, pacing strategies produced divergent effects on ventricular activation velocity (**Figure 8**, right panel). RVSP reduced unpaced AVM, as reflected by a lower RMSE, by enhancing FAP synchrony between the ventricles, but it did not fully restore maximal activation velocity in both chambers (**Table 3**). Both ns-LBBP and Anodal+ further improved FAP synchrony, although maximal activation velocities observed under NSR were not reached in either ventricle (**Table 3**). Anodal+ slightly prolonged FAP in the LV; nevertheless, AVM approached control levels for both modalities. In RBBP, neither ns-RBBP nor s-RBBP fully restored FAP synchrony across both ventricles. ns-RBBP recovered maximal RV activation velocity, but LV exhibited prolonged FAP with reduced peak velocity, whereas s-RBBP maintained LV FAP duration and peak velocity. Both RBBP modalities increased AVM beyond unpaced RBBB, exceeding values observed with the native conduction delay.

**Table 3:** Maximum and mean ventricular activation velocities (%/ms) in LV and RV measured under NSR, RBBB, and corresponding pacing modalities, corresponding to **Figure 7**.

	LV		RV	
Condition	Mean	Max	Mean	Max
NSR	1.60	3.20	1.50	3.30
RBBB	1.60	3.20	1.0	2.40
RVSP	1.20	2.50	1.10	2.80
ns-LBBP	1.25	2.70	1.10	2.80
Anodal+	1.35	2.50	1.10	2.80
ns-RBBP	1.20	2.40	1.20	3.80
s-RBBP	1.50	3.10	1.30	2.80

Maximum velocity denotes the peak activation rate reached during depolarisation, whereas mean velocity represents the average activation rate over the entire depolarisation period.

## 4 Discussion

### 4.1 Principal findings

Using an anatomically detailed computational heart model incorporating a physiologically realistic cardiac conduction system, this study simulated electrophysiological behaviour under controlled bundle branch block conditions to enable a systematic comparison of widely used pacing strategies in the context of CRT. Specifically, CSP modalities were evaluated alongside and RVSP under both LBBB and RBBB, with particular emphasis on how apparent resynchronisation efficacy depends on the assessment metric employed. By integrating surface ECG markers with detailed ventricular activation mapping and activation velocity analysis, the results demonstrate that pacing strategies may appear favourable in one evaluative domain while exhibiting residual or even opposing abnormalities in others. This multi-domain framework reveals mechanistic trade-offs between pacing approaches that would remain obscured if resynchronisation were assessed using a single class of metrics, underscoring the necessity of comprehensive evaluation when interpreting pacing efficacy.

### 4.2 Metric-dependent assessment of resynchronisation across pacing strategies

Despite RVSP being developed to overcome limitations of conventional right ventricular apical pacing by engaging septal myocardium and being clinically associated with QRS shortening (Victor et al., 2006; Worsnick et al., 2018; Zhuang et al., 2018), in the present study RVSP did not achieve substantial QRS shortening relative to other pacing modalities. When applied under both LBBB and RBBB, RVSP produced remarkably similar ECG markers (**Figures 4B, H**) despite fundamentally different underlying activation patterns (**Figure 5C**; **Figure 6C**).

Myocardial capture under RVSP, which resulted in shortening of the H-V interval, was reflected in the activation maps by premature activation of the LV septal region (**Figure 5C**; **Figure 6C**). Although surface ECG markers (V1RWPT and V6RWPT) indicated improved activation timing in the ventricle affected by conduction delay, they did not capture the patchy conduction delay evident in the activation maps (**Figure 5C**; **Figure 6C**). Despite RVSP achieving a V6RWPT close to the NSR reference under LBBB, the corresponding activation velocity profile (**Figure 8**) remained prolonged and slowed. Similarly, although V6V1II was near physiological (13 ms) under paced LBBB, pronounced FAP dyssynchrony persisted, highlighting residual electrical desynchronisation not captured by ECG timing markers alone.

BiVP is well established for delivering CRT in patients with LV systolic dysfunction and prolonged QRSd (Cleland et al., 2013). Nevertheless, response remains heterogeneous, often due to absent intrinsic LV dyssynchrony, suboptimal lead placement–particularly in scarred myocardium–and inappropriate device programming (Bleeker et al., 2006; Murphy et al., 2006; Bilchick et al., 2014). In this study, we found that BiVP, even with optimised interventricular delay (LV-first offset +20 ms) as recommended (Porciani et al., 2005; Ypenburg et al., 2008), produced noteworthy effects.

Activation maps revealed pronounced non-physiological features, including reversed LV activation, delayed septal engagement, and displacement of the LAS from the lateral wall to the anteroseptal region (**Figure 5D**). Despite this, ECG markers under BiVP were comparable to NSR (**Figure 4C**), with only relatively prolonged QRSd reflecting non-physiological epicardial-to-endocardial LV activation. Consistent with these ECG findings, activation velocity profiles closely approximated NSR, with restored peak velocities and synchrony of the FAP (**Figure 8**).

Within the spectrum of CSP strategies, LVSP represents a variant of left bundle branch area pacing (Burri et al., 2023; Diaz et al., 2024). Compared with LBBP, it has been associated with longer left ventricular activation time and a wider paced QRSd (Diaz et al., 2024). Under LBBB, LVSP programming enables prioritisation of either optimisation of the H-V interval or shortening of left ventricular activation time (LVAT), with improvement in one typically occurring at the expense of the other (Vijayaraman et al., 2019; Domenichini et al., 2023). In the present study, LVSP resulted in prolonged LVAT (**Figure 4D**), reflected by an increased V6RWPT. This finding closely aligned with activation maps, which demonstrated discrete regions of residual conduction delay within the LV (**Figure 5**). Despite V6V1II values under LVSP exceeding NSR by more than 30%, ventricular FAP remained well synchronised (**Figure 8**), leading to a markedly reduced AVM.

LBBP has demonstrated variable efficacy across studies. Several reports describe a strong capacity to restore homogeneous LV activation and mechanical synchrony, in some cases exceeding the benefits achieved with BiVP in patients with LBBB (Huang et al., 2017; Cano and Vijayaraman, 2021; Ponnusamy and Vijayaraman, 2021; Wang et al., 2021; Diaz et al., 2023). In contrast, other studies have reported less favourable outcomes, including limited haemodynamic improvement and lower implantation success rates (Vijayaraman et al., 2023; Jastrzębski et al., 2024). Beyond LBBB, growing evidence supports the role of LBBP in RBBB, where QRS narrowing, reduced interventricular mechanical delay, and improved LV ejection fraction have been reported (Vijayaraman et al., 2022; Mirolo et al., 2023). In this context, both Anodal+ and ns-LBBP have been proposed as viable strategies to improve ventricular synchrony in RBBB.

In the simulated LBBB scenario, s-LBBP produced the shortest QRSd among all pacing strategies evaluated, consistent with prior clinical observations (Wang et al., 2020). Both s-LBBP and ns-LBBP effectively reduced LVAT, primarily through shortening of V6RWPT, in agreement with the corresponding activation maps (**Figures 5F,G**). However, important discrepancies emerged when electrical timing metrics were examined alongside activation dynamics. Despite favourable reductions in LVAT, nsLBBP was associated with reduced activation velocity (**Figure 8**), a feature not readily apparent from selected ECG markers alone. Although both forms of LBBP shortened V6V1II relative to LBBB, this was accompanied by reversal of interventricular synchrony (**Figures 4E,F**). For s-LBBP, this manifested as clear FAP dyssynchrony in the activation velocity profiles (**Figure 8**), whereas ns-LBBP preserved more favourable FAP synchrony despite deviations of V6V1II from NSR.

In the presence of RBBB, both ns-LBBP and Anodal+ pacing were associated with shortening of the time to the *R*^′^ deflection and reduction of its amplitude in lead V1, with effects modestly greater under Anodal+. These findings are consistent with prior reports suggesting that dual-site septal activation may partially compensate for intrinsic RV conduction delay (Zhu et al., 2021; Vijayaraman et al., 2022; Curila et al., 2023).

The shortened H-V interval observed with ns-LBBP (**Figure 4I**) was accompanied by earlier septal activation on activation mapping (**Figure 6D**). In contrast, despite greater interventricular septal pre-excitation during Anodal+ pacing (Zhu et al., 2021; Rijks et al., 2024), the apparent H-V interval remained similar to NSR (30 ms; **Figure 4J**). The divergence between ECG markers and activation maps (**Figure 6E**) may be explained by partial cancellation of opposing depolarisation wavefronts originating from the LV pacing site and the RV ring electrode, leading to a semi-isoelectric segment in the lateral ECG leads. Although V6V1II remained prolonged relative to physiological reference values, activation velocity profiles suggested improved synchrony of the FAP between ventricles. Notably, both pacing modalities were associated with prolonged FAP within the LV and reduced peak activation velocity, while V6RWPT remained near physiological levels (approximately 70 ms; **Figures 4J,K**). These features were not readily evident from the selected ECG markers alone, highlighting the limitations of conventional ECG-based metrics in fully capturing underlying activation dynamics.

RBBP represents a CSP variant for patients with RBBB, targeting engagement of the rightsided conduction system (Burri, 2023; Jastrzębski et al., 2023). Similar to LBBP, ventricular activation under RBBP may depend on retrograde engagement of the contralateral bundle branch, potentially resulting in delayed activation of the corresponding ventricle.

In our results, RBBP improved RV activation, as reflected by a reduction in V1RWPT (**Figures 4K,L**). This finding was supported by activation maps demonstrating complete correction under s-RBBP and more modest acceleration with ns-RBBP (**Figures 6F,G**). The H-V interval differed between the two RBBP strategies, being prolonged under s-RBBP (48 ms) and markedly shortened under ns-RBBP (15 ms), while V6RWPT remained prolonged in both cases, consistent with activation map observations (**Figures 6F,G**). Despite its longer V6RWPT, s-RBBP achieved the shortest QRSd among all pacing modalities evaluated in the RBBB setting, reinforcing that QRSd alone is insufficient to comprehensively characterise ventricular resynchronisation (Yu et al., 2003; Haghjoo et al., 2007). Both pacing strategies shifted V6V1II closer to physiological values; however, similar to LBBP, they induced reversed dyssynchrony, which was more pronounced under s-RBBP. In contrast, activation velocity demonstrated greater dyssynchrony under ns-RBBP, with prolonged and slowed FAP within the LV.

These findings underscore that the perceived effectiveness of ventricular pacing strongly depends on the evaluation metric employed. While surface ECG markers suggested favourable outcomes for several strategies, model-derived activation maps and velocity analyses frequently revealed residual non-physiological propagation, incomplete correction of dyssynchrony, or strategy-specific trade-offs. Collectively, this demonstrates that ventricular resynchronisation is inherently multidimensional: reliance on a single metric cannot fully capture resynchronisation efficacy, and patient-specific computational modelling may provide a necessary framework for integrative, non-invasive, multi-metric assessment of pacing efficacy in silico.

### 4.3 Clinical implications

The metric-dependent efficacy observed in this controlled computational setting has important implications for clinical evaluation and optimisation of CRT. Current guidelines predominantly rely on QRSd and morphology for CRT response assessment (Glikson et al., 2025), yet our findings demonstrate that these markers may obscure residual electrical dyssynchrony detectable through alternative assessment modalities (Eltsov et al., 2025). Recent clinical studies using body surface potential mapping and electroanatomical mapping have similarly identified discordance between conventional ECG markers and spatial activation patterns (Rijks et al., 2024; Eltsov et al., 2025).

Activation velocity offers a mechanistic complement to scalar ECG-based timing metrics by capturing the temporal evolution of ventricular depolarization and detecting dyssynchrony not captured by QRS-based measures. Established ECG-based measures, including LV activation time (V6RWPT), RV activation time (V1RWPT), and total activation time approximated by QRS duration, primarily summarise the onset and completion of ventricular depolarisation (Jastrzębski, 2021). As such, these indices represent static or integral descriptors of activation spread and provide limited information on the temporal dynamics of myocardial recruitment (Leinveber et al., 2024).

In contrast, activation velocity characterises the instantaneous rate at which myocardial tissue is depolarised throughout the activation process, offering a dynamic description of ventricular recruitment beyond total activation duration. In recent years, ultra-high-frequency ECG has been introduced as a signal-processing approach that analyses the high-frequency components of the QRS complex to reconstruct the temporal progression of ventricular depolarisation with improved resolution compared to conventional ECG (Jurak et al., 2020). This enables extraction of regional activation timings and more detailed assessment of electrical dyssynchrony. In this context, it is conceptually aligned with activation velocity, as both aim to characterise the temporal dynamics of ventricular recruitment beyond static timing measures.

A similar concept exists in the mechanical domain, where global strain rate derived from speckletracking echocardiography quantifies the rate of myocardial deformation and reflects how rapidly the whole ventricle contracts (Leitman et al., 2004; Risum et al., 2012). In this context, activation velocity may be interpreted as the electrical counterpart of strain rate, extending the concept of time-resolved recruitment to depolarisation dynamics.

Activation velocity is a model-derived quantity and is not directly measurable from routine surface ECG; however, its physiological relevance may be supported through comparison with highdensity electroanatomical mapping and mechanical indices such as strain rate. It should therefore be interpreted as a complementary descriptor within a multi-metric framework.

Similarly, evaluation of the activation sequence provides spatial insight into the order and pattern of ventricular activation, identifying regions of delayed or aberrant propagation that may not be reflected in timing or velocity alone. Integrating activation sequence and velocity with conventional ECG markers could therefore enhance patient-specific pacing optimisation, particularly in complex cases where single-domain evaluation yields ambiguous or incomplete assessments. In this context, computational modelling may support the development of more comprehensive patient-specific CRT evaluation frameworks. Such frameworks may be particularly valuable when combined with spatially resolved electrical assessment methods, including electrocardiographic imaging or electroanatomical mapping, to better characterise the underlying dyssynchrony substrate beyond standard surface ECG. In the longer term, this type of integrated multi-modal approach may help refine patient selection and guide optimisation of pacing strategy, lead location, and device timing in a patientspecific manner.

### 4.4 Limitations and future work

The present study is based on a single patient-specific anatomical model and therefore does not capture the full spectrum of inter-individual variability observed in clinical populations. Structural variations, including differences in ventricular size, fibre architecture, and conduction system morphology, are known to influence baseline activation patterns (Punske et al., 2005; Kariman et al., 2025) and may alter the response to pacing. As the regional distribution of Purkinje–myocardial junctions, particularly on the RV septum, remains uncertain, the subendocardial fast-conducting layer was implemented in both ventricles as a simplified functional representation of rapid endocardial activation, rather than as an explicit anatomical reconstruction of local Purkinje terminal density.

A further limitation is that the simulations were performed in a controlled setting of focal conduction block without concurrent structural myocardial disease. In clinical CRT populations, fibrosis, scar, and heterogeneous tissue remodelling are common and may substantially modify electrical propagation (Massoullié et al., 2019). Such structural abnormalities can introduce regions of conduction slowing or block, alter anisotropic propagation, and thereby affect activation sequence (Campos et al., 2019), potentially influencing the relative efficacy of pacing strategies across different assessment metrics (Zhang et al., 2023). Moreover, retrograde activation of the contralateral bundle branch was permitted in the present model through the proximal His-bundle under controlled focal block conditions. We acknowledge that this represents a modelling assumption and that, *in vivo*, its occurrence is likely to depend on capture site, refractoriness, and the integrity of the proximal His–Purkinje system.

Nevertheless, the central finding of this study, namely that the apparent efficacy of pacing depends on the chosen assessment metric, is expected to remain relevant in more complex pathological substrates. In such settings, conventional ECG markers may show only limited changes, whereas underlying activation dynamics, such as activation velocity, may remain more noticeably altered and are not directly captured by QRS-related surface ECG markers (Rowin et al., 2012; Viriyanukulvong et al., 2024; Ashkir et al., 2025).

Future work will extend this investigation to more complex pathological conditions and incorporate a broader cohort of virtual hearts, including both male and female anatomies, to better capture inter-individual variability and enhance the generalisability of the observations.

The proposed activation velocity metric has not yet been validated against *in vivo* measurements. Future work should therefore focus on systematic validation against independent clinical datasets, including electrical mapping modalities such as electroanatomical mapping or non-invasive electrocardiographic imaging, as well as mechanical indices derived from imaging (e.g., strain or strain rate). Such multimodal validation will be essential to establish the physiological relevance and translational utility of activation velocity as a descriptor of ventricular dyssynchrony.

We acknowledge that the density of Purkinje–myocardial junctions on the RV septum remains debated. In the present model, the subendocardial fast-conducting layer was therefore implemented in both ventricles as a simplified representation of rapid endocardial activation at a moderate conduction velocity, rather than as a direct statement on local Purkinje terminal density.

### 4.5 Translational perspective

CRT aims to restore coordinated ventricular activation to improve cardiac function and outcomes, and therefore depends critically on how resynchronisation is assessed in practice (Foley et al., 2009; Solomon et al., 2010; Thomas et al., 2019; Allison et al., 2022). In silico modelling provides a controlled, risk-free environment in which pacing strategies and assessment metrics can be compared within the same anatomy, complementing clinical studies where such comparisons are typically not feasible (Meiburg et al., 2023).

This computational study shows that reliance on single-domain indices, such as surface ECG markers, may obscure residual dyssynchrony or non-physiological activation patterns, highlighting the need for a multimodal, patient-specific evaluation framework in silico. Such discrepancies are likely to be amplified in clinical populations, where conduction abnormalities are compounded by structural remodelling, fibrosis, and scars (Strauss et al., 2013; Liu et al., 2024).

From a broader clinical perspective, these findings indicate that inter-individual variability may influence how resynchronisation efficacy is interpreted depending on the chosen metric. Consequently, pacing strategies that appear favourable based on conventional ECG criteria may not consistently correspond to underlying activation patterns across heterogeneous patient populations, supporting the need for integrative, multi-metric assessment in clinical decision-making. Clinical translation will require prospective validation of these mechanistic descriptors against multimodal *in vivo* measurements (e.g., electroanatomical mapping or non-invasive electrocardiographic imaging) and demonstration of incremental value beyond standard ECG markers for predicting acute haemodynamic response and longer-term remodelling.

## 5 Conclusions

This computational study provides a systematic comparison of CSP strategies using an anatomically and biophysically detailed representation of the human heart. Assessment of pacing efficacy proved highly metric-dependent, with conventional ECG markers providing incomplete characterization of electrical resynchronisation. The novel activation velocity metric revealed interventricular dyssynchrony patterns not captured by standard QRS-based measures. These findings challenge reliance on single electrical markers and support the development of multimodal evaluation frameworks for patient-specific pacing optimization.

## Data Availability

The raw data supporting the conclusions of this article will be made available by the authors, without undue reservation.

## Appendix. A scoring framework for comparative evaluation of pacing strategies

**Table:** Table A.1 Scoring scheme for ECGs.

ECG metric	NSR reference (ms)	Scoring criteria
H-V interval	30	21–39 ms (± 30%)
QRSd	75	53–98 ms (± 30%)
V1RWPT	61	42–80 ms (± 30%)
V6RWPT	73	51–95 ms (± 30%)
V6V1II	12	8–16 ms (± 30%)

ECG-based scoring framework for comparison of pacing strategies related to **Figure 4**. Physiological reference values were defined from NSR. For each ECG metric, one star was assigned when the value obtained under a given pacing modality fell within ±30% of the corresponding physiological reference range; values outside this range received no star.

**Table:** Table A.2 Scoring scheme for activation maps.

Map type	Feature	Star deduction
LV Endocardial	Partial conduction delay	-1 star
LV Endocardial	Moderate global delay	-2 stars
LV Endocardial	Severe global delay	-4 stars
LV Endocardial	Premature activation	-1 star
LV Endocardial	Discordant activation sequence	-4 stars
BiV Epicardial	Partial delay/incomplete correction in one ventricle	-1 star
BiV Epicardial	Global delay in one ventricle	-2 stars
BiV Epicardial	Premature activation in one ventricle	-1 star
BiV Epicardial	Discordant activation sequence in one ventricle	-2 stars

ChT, Scoring framework for LV endocardial and BiV epicardial activation maps related to **Figures 5**, **6**. Activation sequences were compared to physiological baseline (NSR). Stars were deducted according to the type and severity of conduction delay or pattern discordance.

**Table:** Table A.3 Scoring scheme for activation velocities.

Feature	Deviation from NSR	Rating impact
FAP duration	Prolonged FAP in LV or RV	-1 star per ventricle
Peak activation velocity	Reduced maximum velocity in LV or RV	-1 star per ventricle
Interventricular dyssynchrony	LV–RV FAP temporal mismatch	-1 star

Scoring framework for ventricular activation velocity profiles **Figure 8**. Metrics were evaluated relative to physiological activation dynamics obtained under NSR, with stepwise star deductions applied for deviations indicative of impaired conduction or interventricular dyssynchrony.
